# “Ranolaziodarone”—A Synergism You Should Not Miss

**DOI:** 10.19102/icrm.2021.120303

**Published:** 2021-03-15

**Authors:** James A. Reiffel

**Affiliations:** ^1^Electrophysiology Section, Division of Cardiology, Department of Medicine, Columbia University, New York, NY, USA

**Keywords:** Amiodarone, antiarrhythmic drugs, atrial fibrillation, ranolazine, dronedarone

In this issue of *The Journal of Innovations in Cardiac Rhythm Management*, Aidonidis et al.^1^ report on the effects of ranolazine and its combination with amiodarone on rapid pacing–induced reentrant atrial tachycardia in rabbits. In their study, the authors observed that, (1) in the dose given, ranolazine did not abolish the inducibility of atrial tachycardia in any of the eight rabbits studied, but did prolong its cycle length; (2) supplemental amiodarone, in the dose given, increased the atrial tachycardia cycle length further, without abolishing reinducibility; (3) the more pronounced slowing of the tachycardia with the combined regimen was associated with spontaneous termination; and (4) ranolazine prolonged atrial postrepolarization refractoriness, which was further prolonged when amiodarone was added. The authors commented on the possible clinical relevance of these observations by noting that “collectively, although these experimental data cannot be directly extrapolated to human subjects with structural heart disease, their synergistic electrophysiologic actions support the contention that a combination of ranolazine and amiodarone for the termination and prevention of sustained atrial tachycardia might be better than either of these agents alone.”

While it is a big leap (or, should I say, hop) from eight rabbits to the management of atrial tachyarrhythmias in humans—including, most importantly, atrial fibrillation (AF), mankind’s most common and troublesome atrial tachyarrhythmia—the use of a combination of antiarrhythmic drugs (AADs) to treat arrhythmias in humans (and other species) is not a new concept. Moreover, neither is the use of ranolazine combined with amiodarone, or its derivative, dronedarone. In reality, AADs in combination with each other have been used for difficult-to-manage arrhythmias for decades. For example, prior to the development of the implantable cardioverter-defibrillator, patients with refractory ventricular arrhythmias were either managed with empiric amiodarone or with AADs in combination, such as quinidine plus mexiletine, with moderate success.^2,3^ Similarly, beyond their use to achieve added efficacy, when the tolerance of a full dose of an AAD was an issue despite its efficacy, lower-dose combinations of AADs have been tried, such as quinidine plus disopyramide, with each offsetting the other’s gastrointestinal side effects.^4,5^

With respect to ranolazine in particular and its use as an antiarrhythmic agent in combination with another AAD, the report by Aidonidis et al.^1^ is not unique with regard to the combination of ranolazine with amiodarone nor in its evaluation in a nonhuman species. Like amiodarone and dronedarone, ranolazine is a multichannel blocker that has electrophysiologic effects and efficacy in patients with cardiac arrhythmias, including AF.^6,7^ The specific electrophysiologic effects of each of these agents, however, are different. In a model assessing ranolazine plus amiodarone in canine atria, the combination produced a synergistic use-dependent depression of sodium current–dependent parameters, leading to a potent effect of the drug combination in preventing the induction of AF in association with both a marked increase in postrepolarization refractoriness and a reduction of triggered activity in pulmonary vein sleeves.^8^ In additional in vitro investigations, ranolazine again showed efficacy against AF. In a 30-rabbit study of ranolazine added to amiodarone (n = 10), dronedarone (n = 10), or placebo, followed by the administration of intravenous sotalol (n = 10), the addition of ranolazine led to an added increase in the atrial effective refractory period and postrepolarization refractoriness beyond that achieved with the class III AADs alone, together with an increase in the interatrial conduction time. Amiodarone-pretreated hearts showed a lower incidence of AF, which was further reduced with the addition of ranolazine. Dronedarone or intravenous sotalol did not suppress AF, whereas they did when ranolazine was added.^9^ Similar findings were made in a rabbit heart study of ranolazine added to selective inhibitors of the sodium–calcium exchanger.^10^

With respect to studies in humans with AF, multiple reports have been published verifying the increased efficacy for the termination of and/or the prevention of AF.^11–17^ Most of these studies involved patients who were referred for pharmacologic cardioversion of recent-onset AF or patients with AF following cardiac surgery. In each of them, amiodarone was acutely administered intravenously, while ranolazine was added orally. In one,^16^ the combination worked more rapidly in patients with reduced left ventricular ejection fraction values than in those with preserved left ventricular ejection fraction values.

In a manner similar to in the human studies with amiodarone, ranolazine has been studied in combination with dronedarone, where the combination has again been shown to have enhanced efficacy. Here, too, the studies performed have included both in vitro and in vivo investigations. In a canine isolated coronary-perfused atrial and pulmonary vein preparation, low concentrations of intravenous ranolazine and dronedarone, when perfused separately, produced relatively weak electrophysiological effects and weak suppression of AF. However, when combined, they exerted “potent synergistic effects resulting in atrial-selective depression of sodium channel-dependent parameters and effective suppression of AF.”^18^ Similarly, using intravenous ranolazine and dronedarone, this time in an open-chest Yorkshire pig model with induced acute ischemia, neither agent blunted the ischemia-induced reduction in AF threshold, yet both in combination were effective in blunting both the AF duration (p < 0.03) and inducibility (p = 0.012).^19^ Additionally, in patch-clamp experiments with atrial myocytes from patients in sinus rhythm obtained surgically, synergistic electrophysiologic effects were demonstrated.^20^ Here, ranolazine combined with low-dose dronedarone showed atrial action-potential duration prolongation, cellular hyper-repolarization, and reduced sarcoplasmic reticulum calcium current leak.

Notably, this latter study was performed to help explain the results of the HARMONY trial,^21^ which tested midrange ranolazine (750 mg twice daily), midrange dronedarone (225 mg twice daily), ranolazine (750 mg twice daily) combined with low-dose dronedarone (150 mg twice daily), and ranolazine 750 mg twice daily combined with dronedarone 225 mg twice daily versus placebo in the suppression of paroxysmal AF. Study participants were all patients (n = 134) with paroxysmal AF demonstrated prior to the initiation of drug administration and with previously implanted permanent pacemakers capable of being interrogated to demonstrate AF burden. The study revealed no efficacy of 225 mg of dronedarone twice daily versus placebo in reducing the AF burden but did report a progressive, absolute decrease in AF burden with 750 mg of ranolazine twice daily (though p = 0.493), 750 mg of ranolazine twice daily plus 150 mg of dronedarone twice daily (p = 0.072), and 750 mg of ranolazine twice daily plus 225 mg of dronedarone twice daily (p = 0.008). The tolerance and safety of the combination were excellent. It was hoped following the success of the trial that further studies would be performed, leading to approval by the United States Food and Drug Administration (FDA) of the twice-daily combination of 750 mg of ranolazine plus 225 mg dronedarone (both are doses that are not currently marketed individually); however, changes within the sponsoring company, Gilead Sciences (Foster City, CA, USA), and patent-life issues as well as FDA concerns about the size of the trial have thus far prevented subsequent investigations from taking place. Nonetheless, both drugs are available individually in the United States and have been used in combination at FDA-approved doses by physicians at our center.^22–24^

Finally, in addition to its combination with amiodarone and dronedarone, ranolazine has also been studied in in vitro and in vivo models in combination with the administration of dofetilide in both a human case study and in horses, where beneficial effects on AF vulnerability, duration, and conversion have been similarly observed.

Accordingly, the report by Aidonidis et al.^1^ can be added to a growing list of studies that demonstrate the atrial antiarrhythmic benefits of ranolazine both alone and in combination with other AADs, including, most particularly, amiodarone and dronedarone. For AF, a particularly bothersome arrhythmia for humans—both male and female patients alike—and for their caregivers, this unique drug (ranolazine) and combination approach (which might be termed “ranolaziodarone”) could be particularly useful.
